# Atrial Fibrillation Detected by Implantable Monitor in Embolic Stroke of Undetermined Source: A New Clinical Entity

**DOI:** 10.3390/jcm11195740

**Published:** 2022-09-28

**Authors:** Salomé Snyman, Elena Seder, Marc David-Muller, Victor Klein, Emilie Doche, Laurent Suissa, Jean-Claude Deharo, Emmanuelle Robinet-Borgomano, Baptiste Maille

**Affiliations:** 1 Stroke Unit, University Hospital of Marseille, La Timone 264, Rue Saint-Pierre, 13005 Marseille, France; 2 Rhythmology Unit, Cardiology Department, University Hospital of Marseille, La Timone 264, Rue Saint-Pierre, 13005 Marseille, France; 3 Centre de Recherche en Cardiovasculaire et Nutrition (C2VN), 27, Bd Jean-Moulin, 13005 Marseille, France; 4 Marseille School of Medicine, 27, Bd Jean-Moulin, 13005 Marseille, France

**Keywords:** atrial fibrillation, ESUS, stroke, cardiac monitoring, stroke prevention

## Abstract

Background: High incidence of covert paroxysmal atrial fibrillation (CPAF) detected by an implantable cardiac monitor (ICM) is expected in embolic stroke of undetermined source (ESUS) patients. This study aimed to determine the CPAF rate in an ESUS cohort using ICMs and compare stroke characteristics of patients with CPAF to those with known or inpatient-diagnosed AF (KIDAF). Methods: ESUS patients with ICMs were enrolled. ESUS diagnosis was defined as a non-lacunar stroke in the absence of symptomatic atherosclerotic stenosis (≥50%), no major-risk cardioembolic source, and no other specific cause. ESUS characteristics of patients with CPAF were compared to ESUS patients without CPAF and to KIDAF stroke patients. Results: During the median follow-up of 476 (371–615) days, CPAF was newly detected in 38/163 (23.31%) patients within 236 (115.50–510.75) days after the stroke. CPAF was independently associated to older age, coronaropathy, left atrial dilation, and atrial hyperexcitability, but not to stroke severity. Compared to KIDAF strokes, ESUS with CPAF had lower rates of proximal occlusion leading to milder clinical severity (NIHSS: 3.00 (1.00–8.25) vs. 14.50 (6.00–21.00)). Conclusions: Our study revealed a high proportion of CPAF in ESUS. We highlight that CPAF is a distinct clinical entity compared to KIDAF based on differences in stroke characteristics and AF diagnosis temporality.

## 1. Introduction

In 2014, the clinical construct of “embolic stroke of undetermined source” (ESUS) was introduced by Hart et al. to describe patients with non-lacunar ischemic strokes and no convincing etiology, and in whom an underlying embolic mechanism was suspected [1]. ESUS involves approximately 17% of all ischemic strokes with an annualized recurrence rate of 4–5% [2,3,4].

Covert Paroxysmal Atrial Fibrillation (CPAF) was conceived initially as the most important underlying source in ESUS, as atrial fibrillation (AF) was detected in up to 24% of cryptogenic strokes [5,6,7]. In recent years, intensive cardiac monitoring including with Implantable Cardiac Monitors (ICMs) after stroke was associated with higher AF diagnoses [5,6,7], and such monitoring is now recommended after cryptogenic strokes, including ESUS [8].

In the setting of presumed high incidence of CPAF in ESUS, the empiric use of oral anticoagulants instead of the standard aspirin treatment for secondary prevention in ESUS has been investigated in several studies [3,4,9,10]. The first trials [3,4] failed to prove a superiority of oral anticoagulation and raised questions about CPAF diagnosis in ESUS: its incidence, its clinical significance, and the diagnostic workup that should be undertaken to detect it.

The aims of this study were to determine the rate of CPAF in ESUS using ICMs, to describe the clinico-radiological patterns in this group, and to compare their characteristics with stroke patients presenting known or inpatient-diagnosed AF (KIDAF).

## 2. Materials and Methods

### 2.1. Study Population

We studied a population of ESUS patients admitted to the neurovascular unit of the University Hospital of Marseille who had undergone long-term cardiac monitoring. We enrolled patients of 18 years and above who had been hospitalized for an ischemic stroke documented by cerebral imaging—Computed Tomography (CT) or Magnetic Resonance Imaging (MRI)—and had undergone long-term cardiac monitoring with an ICM by the hospital’s cardiologic department between January 2019 and December 2021. In our hospital, cardiac monitor implantation is approved after a concertation meeting between cardiologists and neurologists. Patients were included in the study if their ESUS diagnosis had been adjudicated by a vascular neurologist after etiological explorations, and if cardiac monitoring of at least 3 months was available.

ESUS diagnosis was defined according to Hart et al. [1] as a stroke detected by CT or MRI that is non-lacunar, in the absence of extracranial or intracranial atherosclerosis causing ≥50% luminal stenosis in arteries supplying the area of ischemia, with no major-risk cardioembolic source and no other specific cause of stroke identified (e.g., arteritis, dissection, migraine, vasospasm, and drug misuse). ESUS diagnosis required a minimal diagnostic assessment as suggested by Hart et al. [1] of a 12-lead electrocardiogram (ECG), a transthoracic echocardiography, cardiac monitoring for 24 h with automated rhythm detection, and imaging of both the extracranial and intracranial arteries supplying the area of brain ischemia (catheter, MR, or CT angiography, or cervical duplex plus transcranial doppler ultrasonography).

In order to compare the clinico-radiological patterns of ESUS with CPAF diagnosis via ICM, to those of strokes with KIDAF, we formed a second cohort of patients who presented strokes with KIDAF in a similar time period using the registry of the Stroke Unit of the University Hospital of Marseille. To form this cohort, all consecutive patients of 18 years and above admitted to the Stroke Unit for a documented ischemic stroke, and for whom stroke etiology was imputed to AF during the index hospitalization, were included. AF diagnosis was either withheld on a history of AF, on admission ECG, on prolonged ECG telemetry during hospitalization stay, or on the 24-h Holter ECG.

The trial protocol was approved by the Institutional Review Board of Marseille and was conducted according to the principle of the Declaration of Helsinki.

### 2.2. Data Collection

Data was collected in the anonymized registry of the Stroke Unit of the University Hospital of Marseille. The registry was filled out by trained vascular neurologists of the Stroke Unit. For each patient, demographic information, medical history, anterior medical treatment, and initial clinico-radiologic stroke characteristics were reported. Clinical stroke severity was assessed by the initial NIH Stroke Scale (NIHSS). Symptomatic arterial occlusion, apprehended by CT or Magnetic Resonance Angiography (MRA), as well as the use of a revascularization treatment (IV-thrombolysis or mechanical thrombectomy) were recorded in the data base.

Cardiologic evaluation was apprehended through morphological, electrophysiological, and biological explorations. The minimal cardiologic evaluations, consisting in a 12-lead ECG, 24-h Holter ECG, and a transthoracic echocardiography, were reviewed by a trained cardiologist of the University Hospital of Marseille and were recorded in the registry. Left atrial dilatation (LAD) was defined as an indexed volume >34 mL/m^2^ [11]. Atrial hyperexcitability was defined as frequent premature atrial complex (more than 400 on 24-h Holter ECG) and/or repetitive atrial activity (from 3 repetitive atrial complexes lasting less than 30 s) [12]. Biological evaluations were performed in the hospital laboratory and included Nt-proBNP (ng/L), US-troponin (ng/L), and TSH (mUI/L) levels.

Some of the post-stroke AF predictive scores of the literature such as CHA2DS2VASc [13], STAF [14], AS5F [15], and HAVOC [16] were calculated for each patient.

The trained neurologists also categorized the stroke phenotype using the ASCOD (A: atherosclerosis; S: small-vessel disease; C: cardiac pathology; O: other causes; D: dissection) classification [17]. ASCOD phenotyping assigns a degree of likelihood of a causal relationship with each potential disease encountered in ischemic stroke (1 for potentially causal, 2 for uncertain causality, 3 for unlikely causality but the disease is present, 0 for absence of disease, and 9 for insufficient workup to rule out the disease).

### 2.3. Atrial Fibrillation Diagnosis

AF was defined as an absence of distinct repeating P-waves with anarchic atrial activations and irregular R–R intervals [18]. In the ESUS group, CPAF diagnosis was sustained in the occurrence of a first episode of AF > 30 s recorded on an ICM. All patients were observed by remote monitoring. All arrhythmic events were manually reviewed and verified by a trained cardiologist. Similarly, atrial tachycardia and atrial flutter were managed as AF episodes. ICM model choice was at the cardiologist’s discretion. In this study, all ICMs were St Jude Confirm RX^®^ or MEDTRONIC Reveal LNQ^®^ models.

In the stroke with KIDAF group, AF diagnosis was adjudicated by a trained cardiologist in the presence of a history of AF, or of an AF episode >30 s on the admission ECG, prolonged ECG monitoring during hospitalization, or on the 24-h Holter ECG.

### 2.4. Statiscal Analysis

Continuous variables are presented accordingly as the median with interquartile range (IQR). Categorical variables are presented as absolute numbers (%). A threshold value was established for continuous variables using a ROC curve at the Youden plot. To determine the statistically significant differences in clinical and paraclinical variables between both groups analyzed, univariate analysis was carried out using the χ^2^ test for comparison of discontinuous variables and the Wilcoxon–Mann–Whitney test for continuous variables. A threshold of *p*-value ≤ 0.05 was considered as significant. Non-redundant variables with *p*-value ≤ 0.05 in univariate analysis were included in a backward stepwise logistic regression. Statistical analyses were conducted using the STATA 10.0 statistical package.

## 3. Results

### 3.1. Description of ESUS Population

Two hundred and forty-nine patients with ischemic stroke benefited from ICM implantation between January 2019 and December 2021. Eighty-six patients were excluded from the analysis (27 patients did not meet ESUS criteria, 45 patients were excluded because of missing data, and 14 patients did not have an ischemic stroke documented). In total, 163 ESUS patients fitted with an ICM were included in the statistical analysis. Demographic characteristics, medical history, and initial stroke characteristics are described in Table 1.

The median age was 69.00 years old IQR (61.00–76.00) and 92/163 (56.44%) patients were men. A total of 93/163 (57.06%) patients had a medical history of high blood pressure, 29/163 (17.79%) had a history of diabetes, and 48/163 (29.45%) had a history of an ischemic stroke of Transient Ischemic Attack (TIA). The median CHA2DS2-VASc score was 4.00 IQR (3.00–5.00) and 98/163 (60.12%) patients presented grade 2 or 3 atherothrombosis according to the ASCOD phenotyping. The median initial NIHSS score was 4 IQR (1.50–9.00).

### 3.2. Proportion of AF Detected on ICM in ESUS

**Figure 1:**
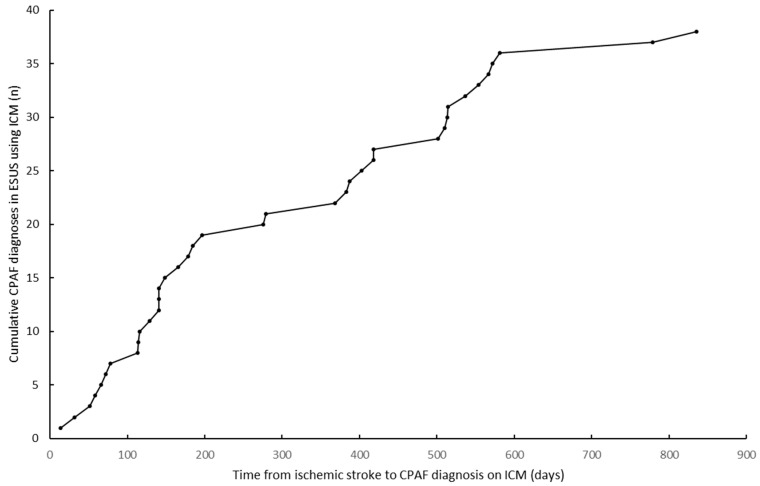
Time from ischemic stroke to CPAF diagnosis in ESUS using ICM.

CPAF was newly detected by ICM in 38/163 patients (23.31%) during the follow-up period, with a median time from the stroke incident to the first AF episode of 236 days IQR (115.5–510.75). Figure 1 shows the cumulative CPAF diagnoses according to the time from stroke onset. The median duration of cardiac monitoring was 476 days IQR (371–615) and the median time between the stroke incident and ICM implantation was 113 days IQR (33.17–246.14).

### 3.3. Characteristics of ESUS Patients with CPAF Detected on ICM

There was no significant difference between ESUS patients with CPAF detection on ICM, and those without CPAF detection in terms of clinico-radiological characteristics (initial NIHSS score, symptomatic arterial occlusion rate, revascularization treatment use) (Table 1). Patients for whom CPAF was detected were significantly older (72.50 years old IQR (68.25–78.00) vs. 67.00 IQR (57.00–76.00)) and were more frequently associated with a history of ischemic coronaropathy (8/38 (21.05%) vs. 11/125 (8.80%)), cervical or intracranial atherothrombosis (30/38 (78.95%) vs. 68/125 (54.40%)), and the use of statins (15/38 (39.47%) vs. 23/125 (18.40%)) and beta blockers (12/38 (31.58%) vs. 20/125 (16.00%)) in usual treatment.

Cardiologic explorations showed that CPAF detection was significantly associated with LAD on echography (25/34 (73.53%) vs. 47/107 (43.93%)), as well as with atrial hyperexcitability on the 24-h Holter ECG (26/121 (21.49%) vs. 18/36 (50.00%)). NT pro-BNP levels at admission were higher in the CPAF detection group although it did not reach statistical significance (*p* = 0.100). HS-troponin levels were significantly associated with CPAF detection (17.00 IQR (10.50–26.00) vs. 11.00 IQR (7.00–17.75)).

All AF prediction scores (CHA2D2S-VASc, STAF, HAVOC, and AS5F) were significantly higher in the CPAF detection group.

Age (OR (/1 y): 1.05 95%CI (1.00–1.10)), history of coronaropathy (OR: 3.70 95%CI (1.01–13.53)), LAD on echography (OR: 2.98 95%CI (1.11–8.01)), and atrial hyperexcitability (OR: 2.83 95%CI (1.05–7.62)) were independently correlated with CPAF detection.

### 3.4. Comparison of ESUS Patients with CPAF Diagnosed by ICM and Stroke Patients with KIDAF

**Figure 2:**
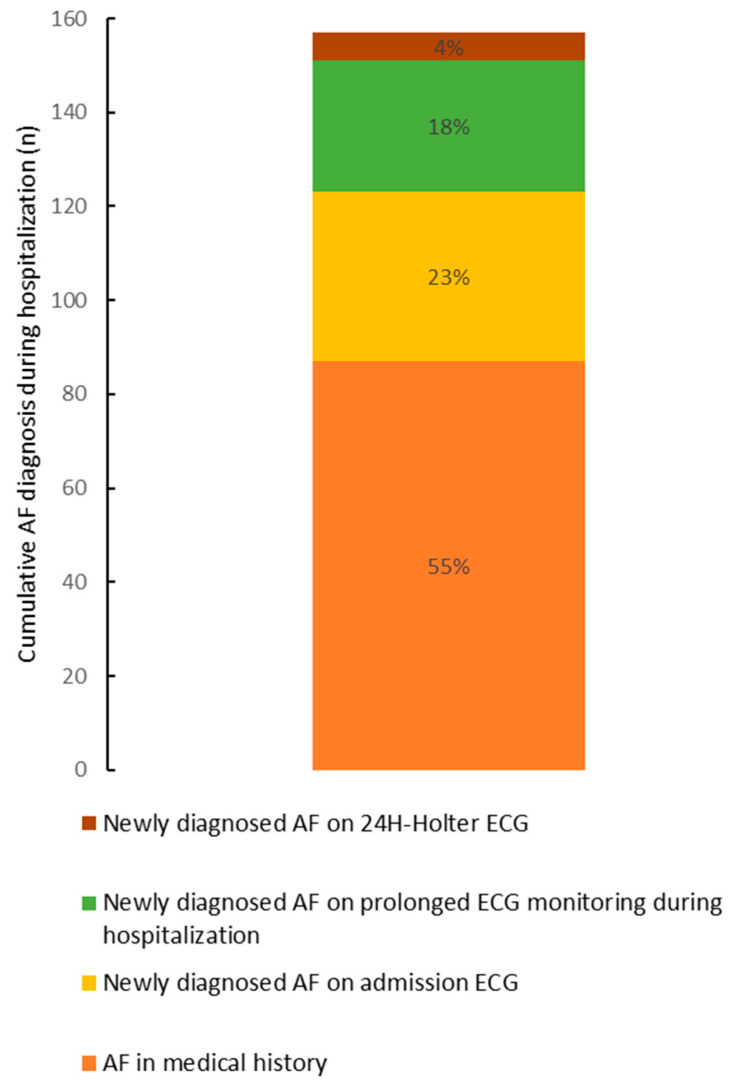
AF diagnosis modalities for KIDAF.

Consecutive patients with ischemic stroke and etiologic diagnosis imputed to AF based on a history of AF or an inpatient AF diagnosis were compared with ESUS patients in whom CPAF had been detected by ICM (Table 2). The modalities for KIDAF diagnosis during the hospitalization are represented in Figure 2.

**Figure 3:**
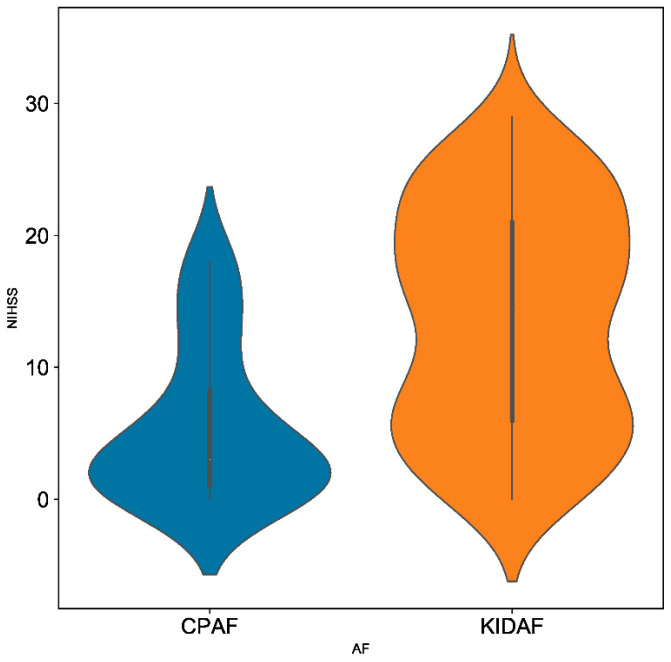
Violin representation of clinical stroke severity (NIHSS) according to CPAF and KIDAF patients.

ESUS patients with CPAF detected by ICM presented less severe strokes than patients with KIDAF, as illustrated by the significantly lower initial NIHSS score in this group (3.00 IQR (1.00–8.25) vs. 14.50 IQR (6.00–21.00)) (Figure 3). A lower prevalence of symptomatic arterial occlusions was also observed, as well as a lower proportion of large vessel occlusions, which was apprehended by the rate of mechanical thrombectomy performed (5/38 (13.16%) vs. 56/157 (35.67%)).

Patients with CPAF detected by ICM were significantly younger than patients with KIDAF. Regarding cardiologic features, LAD occurrence did not differ between the two groups; however, left ventricular dysfunction was more frequent in the KIDAF group (39/122 (31.97%) vs. 4/38 (10.53%)). NT pro-BNP levels were significantly higher in the KIDAF group compared with the CPAF detected by ICM group.

The thromboembolic risk of AF apprehended by the CHA2D2S-VASc score was significantly higher in the KIDAF group. Among the predictive scores of AF occurrence, only AS5F was significantly higher in KIDAF.

## 4. Discussion

In our study, covert paroxysmal AF (CPAF) was newly diagnosed in ≈23% ESUS patients based on intensive cardiac monitoring via an ICM. Interestingly, the clinico-radiological severity of ESUS with CPAF was significantly different compared to KIDAF-related strokes, reinforcing the idea that CPAF and KIDAF are two different clinical entities.

### 4.1. High Rate of CPAF in ESUS

This study was conducted on an ESUS cohort with typical features [19,20]. Indeed, in accordance with our results, ESUS patients are described in the literature as younger, with fewer cardiovascular risk factors and lower stroke severity than non-ESUS patients. CPAF, presumed to be the main cause of ESUS, was newly detected in almost a quarter of these patients using intensive cardiac monitoring by ICM within 236 days IQR (115.5–510.75) from ESUS. This result is consistent with previous studies [21,22,23] and supports the high rate of AF diagnosis when using ICM on ESUS patients. Indeed, in ESUS cohorts without systematic use of intensive cardiac monitoring, CPAF diagnosis rates were respectively 3.4% and 7.5% in the NAVIGATE-ESUS and RE-SPECT ESUS studies [3,4]. In the ATTICUS trial [9], the detection rate of CPAF by ICM in ESUS patients (23%) was similar to our results. In our study, it is not surprising to find that AF risk factors such as age, coronaropathy history, left atrial dilatation, and atrial hyperexcitability were independently associated with CPAF diagnosis [21,22,23,24]. Interestingly, clinical severity assessed by the NIHSS scale was not a predictor of CPAF diagnosis in ESUS patients. AF-related strokes are traditionally associated with a higher NIHSS at stroke admission than naïve-AF strokes [25]. This explains why NIHSS and age are common parameters in post-stroke AF prediction scores [14,15,16]. The STAF score [14], which includes additionally left atrial dilatation criteria, was the best CPAF predictor score in ESUS among the other scores evaluated in this study.

### 4.2. Clinico-Radiological Patterns of Stroke Patients with CPAF or KIDAF Are Different

Our results regarding the different clinico-radiological patterns observed when comparing ESUS with CPAF detected by ICM and strokes with KIDAF support the argument that CPAF and KIDAF are two different entities. Interestingly, the comparison of these two groups of patients revealed lower rates of proximal arterial occlusion (thrombectomy performed in 13.16% vs. 35.67%), leading to a milder clinical severity (median initial NIHSS score 3.00 IQR (1.00–8.25) vs. 14.50 IQR (6.00–21.00)) in the ESUS with CPAF group compared to the stroke with KIDAF group. Traditionally, the clinico-radiological profile of an AF-related stroke is associated with clinical severity, related to cerebral infarcts of great volume with proximal arterial occlusions [25,26]. It is necessary to remember that the AF-related stroke description was defined before the generalization of intensive cardiac monitoring and was based on KIDAF-related stroke cohorts.

### 4.3. CPAF and KIDAF: Two Clinical Entities?

The differences in the clinico-radiological patterns observed in stroke patients with CPAF and KIDAF might be explained by two main hypotheses. In the first hypothesis, the absence of a causality link between CPAF and ESUS might be considered based on both stroke clinico-radiological patterns and AF diagnostic temporality. Thus, CPAF incidence in the ESUS population might only be supported by a higher prevalence of the cardiovascular risk factors and cardio-morphological abnormalities underlying AF without presuming a causality link with ESUS [18,24]. The results of the STROKE-AF study [27] support this hypothesis. In the STROKE-AF study [27], implanted cardiac monitoring after a stroke attributed to large or small vessel disease resulted in an AF rate reaching 12% at 12 months. This study thus suggests a high rate of CPAF in an at-risk population, given its cardiovascular comorbidities. The determined etiology of the index stroke as well as the diagnosis temporality of CPAF again question the causality between CPAF and the incident ischemic event. In the second hypothesis, the causality link between CPAF and ESUS is assumed, and CPAF diagnosed by ICM a long time after the stroke might lead to milder stroke severity in the setting of an earlier stage of AF disease. We discussed the fact that at an earlier stage, AF would be the cause of a less voluminous thrombus in the left atrial cavity, explaining the lower rate of large vessel occlusion and consequently, milder stroke severity in CPAF compared to KIDAF patients in our study. In the literature, two examples of clinical entities support this hypothesis. Interestingly, the incidence of KIDAF in TIA is lower than in AF-related strokes [28]. Moreover, growing evidence suggests a consistent association between atrial fibrillation, cognitive impairment, and dementia, independently of clinical stroke [29]. The cognitive impairment purportedly relates to micro emboli, owing to an earlier stage of AF. Early-stage AF is characterized by a lower AF burden, leading to a reduced stroke recurrence risk [30,31]. Even if the AF burden was not assessed in our study, it is assumed that the AF burden is higher when AF-detection is easily performed, such as in KIDAF [30], which is why we chose to regroup known AF and inpatient-diagnosed AF in the same clinically relevant group (KIDAF), opposing late-diagnosed AF.

### 4.4. Therapeutic Challenges in Secondary Stroke Prevention with CPAF and Perspectives

Our study highlights that CPAF and KIDAF are two clinical entities distinguished by several differences such as stroke characteristics, AF diagnosis temporality, and AF burden, questioning the causality link between CPAF and ESUS. As CPAF causality regarding index stroke is associated with the cardioembolic recurrence risk, our results reinforce the hypothesis that CPAF detected by ICM is associated with a lower cardio embolic recurrence risk, as was suggested by Sposato et al. [32,33,34]. In the past decade, the generalization of intensive cardiac monitoring, including ICMs, has given rise to the new concept of AF Detected After Stroke (AFDAS), introduced by Sposato et al. [32,33,34]. These authors suggest that AFDAS, compared with Known AF (KAF) which is diagnosed based on medical history or on a baseline ECG, is distinguished by its stroke recurrence risk, underlying a new entity concept in AFDAS. As discussed above, based on the strong correlation between easy AF diagnosis during the hospitalization stay and AF burden [30], we suggest preferring KIDAF to KAF and to limit the AFDAS definition for incidental AF diagnosis by ICM. This new concept of CPAF was supported by randomized clinical trials in secondary prevention of ESUS [3,4,9]. Despite a high rate of CPAF in ESUS, therapeutic trials have failed to demonstrate the superiority of direct oral anticoagulants (DOA) in a secondary prevention strategy compared with aspirin, even in selected patients with a confirmed high AF proportion, such as in ATTICUS. In these circumstances, the thromboembolic recurrence risk of CPAF detected by ICM is questionable. Further research in stroke secondary prevention is now required, considering CPAF, incidentally diagnosed by ICM, as a new challenging entity [32,33,34]. Even though CPAF detected by ICM leads to the use of direct oral anticoagulant in secondary prevention of stroke [8], the efficiency of this strategy on preventing stroke recurrence is not established [32,33,35]. Randomized clinical trials are now required to answer these major questions in this field of secondary stroke prevention.

### 4.5. Limits

In this monocentric retrospective study, major clinical severity or premature death prevented the classification of some patients as ESUS, as the minimal diagnosis workup requirement had not been fulfilled, possibly leading to an underestimation of clinical severity. Because cardiac monitor implantation was decided in concertation meetings, exhaustivity of ESUS patients in this study is not guaranteed. Additional studies are needed to confirm our results based on the limited cohort of CPAF patients.

## 5. Conclusions

Our study based on intensive cardiac monitoring via ICM use in a population of ESUS patients revealed a high rate of CPAF (≈23%). In addition to different AF diagnosis temporalities, we highlight that CPAF is a distinct clinical entity from KIDAF, given the differences of stroke characteristics. All of these findings raise questions on the cardio embolism risk of CPAF incidentally diagnosed by ICM in ESUS. We call for further research in the field of stroke secondary prevention.

## Figures and Tables

**Table 1:** Characteristics of ESUS population according to CPAF diagnosis on ICM (missing data is in brackets).

	ESUS Patients with ICM (*n* = 163)	CPAF Detected on ICM (*n* = 38)	No CPAF Detected on ICM (*n* = 125)	*p*
**Demographic data**				
Men	92/163 (56.44%)	24/38 (63.16%)	68/125 (54.40%)	0.340
Age (years)	69.00 IQR (61.00–76.00)	72.50 IQR (68.25–78.00)	67.00 IQR (57.00–76.00)	0.002
**Medical history**				
Hypertension	93/163 (57.06%)	25/38 (65.79%)	68/125 (54.40%)	0.214
Diabetes	29/163 (17.79%)	8/38 (21.05%)	21/125 (16.80%)	0.548
Dyslipidemia	80/163 (49.08%)	21/38 (55.26%)	59/125 (47.20%)	0.384
Current smoker	36/163 (22.09%)	6/38 (15.79%)	30/125 (24.00%)	0.285
Ischemic stroke/TIA	48/163 (29.45%)	11/38 (28.95%)	37/125 (29.60%)	0.938
Coronaropathy	19/163 (11.66%)	8/38 (21.05%)	11/125 (8.80%)	0.039
Peripheral arterial disease	12/163 (7.36%)	2/38 (5.26%)	10/125 (8.00%)	0.572
**Treatments on admission**				
Antiaggregant	60/163 (36.81%)	18/38 (47.37%)	42/125 (33.60%)	0.123
Anticoagulant	1/163 (0.61%)	0/38 (0.00%)	1/125 (0.81%)	0.580
Statin	38/163 (23.31%)	15/38 (39.47%)	23/125 (18.40%)	0.007
Antihypertensive	79/163 (48.47%)	20/38 (52.63%)	59/125 (47.20%)	0.557
Beta blockers	32/163 (19.63%)	12/38 (31.58%)	20/125 (16.00%)	0.034
**Baseline stroke characteristics**				
Clinical stroke severity (NIHSS)	4.00 IQR (1.50–9.00) (12)	3.00 IQR (1.00–8.25) (2)	4.00 IQR (2.00–9.00) (10)	0.300
Multi-territorial strokes	14/163 (8.59%)	2/38 (5.26%)	12/125 (9.60%)	0.403
Anterior circulation stroke	115/163 (70.55%)	25/38 (65.79%)	90/125 (72.00%)	0.462
Posterior circulation stroke	51/163 (31.29%)	13/38 (34.21%)	38/125 (30.40%)	0.657
Symptomatic arterial occlusion	58/161 (36.02%)	14/37 (37.84%)	44/124 (35.48%)	0.794
IV-thrombolysis (rt-PA)	46/163 (28.22%)	9/38 (23.68%)	37/125 (29.60%)	0.478
Mechanical thrombectomy	28/163 (17.18%)	5/38 (13.16%)	23/125 (18.40%)	0.453
**Biological Evaluation**				
NT-proBNP (ng/L)	206.00 IQR (87.48–467.38) (21)	350.00 IQR (130.60–654.00) (3)	177.00 IQR (75.80–412.00) (18)	0.100
HS Troponin T(ng/L)	12.00 IQR (7.00–19.00) (22)	17.00 IQR (10.50–26.00) (3)	11.00 IQR (7.00–17.75) (19)	0.011
TSH (mUI/L)	1.90 IQR (1.15–2.69) (19)	2.16 IQR (1.30–2.96) (3)	1.89 IQR (1.14–2.53) (16)	0.383
**Etiological stroke subtypes (ASCOD)**				
Atherothrombosis (grades 2/3)	98/163 (60.12%)	30/38 (78.95%)	68/125 (54.40%)	0.007
Small vessel disease (grades 2/3)	32/163 (19.63%)	7/38 (18.42%)	25/125 (20.00%)	0.830
**ECG characteristics**				
QRS interval (msec)	80.00 IQR (80.00–90.00) (16)	80.00 IQR (80.00–97.50) (4)	80.00 IQR (80.00–90.00) (12)	0.679
Abnormal heart axis	28/151 (18.54%)	6/34 (17.64%)	22/117 (18.80%)	0.879
**24-h Holter Characteristics**				
Atrial hyperexcitability	44/157 (28.03%)	18/36 (50.00%)	26/121 (21.49%)	0.001
Ventricular extrasystoles	6/147 (4.08%)	3/34 (8.82%)	3/113 (2.465%)	0.111
**TTE Characteristics**				
LVEF < 60%	24/158 (15.19%)	4/38 (10.53%)	20/120 (16.67%)	0.358
Left ventricular hypertrophy	41/152 (26.97%)	11/36 (30.56%)	30/116 (25.86%)	0.579
Left atrial dilatation	72/141 (51.06%)	25/34 (73.53%)	47/107 (43.93%)	0.003
Valvulopathies	22/163 (13.50%)	9/38 (23.68%)	13/125 (10.40%)	0.036
**CPAF modalities detection**				
Stroke to 24-h Holter ECG time (d)	4.04 IQR (2.16–8.05) (12)	4.16 IQR (2.25–7.34) (1)	4.02 IQR (2.00–8.21) (11)	0.651
Stroke to ICM implantation time (d)	113.56 IQR (33.17–246.14) (2)	87.23 IQR (25.29–152.97)	127.31 IQR (36.79–272.56) (2)	0.187
ICM follow-up (d)	476.00 IQR (371.00–615.00)	561.50 IQR (434.00–718.25)	448.00 IQR (357.00–539.00)	0.004
**AF predictive scores**				
CHA_2_DS_2_-VASc [13]	4.00 IQR (3.00–5.00)	5.00 IQR (4.00–5.00)	4.00 IQR (3.00–5.00)	0.007
STAF [14]	6.00 IQR (5.00–7.00) (22)	7.00 IQR (5.00–7.00) (3)	5.00 IQR (5.00–7.00) (19)	<0.001
HAVOC [16]	2.00 IQR (1.00–4.00)	4.00 IQR (2.00–4.00)	2.00 IQR (1.00–4.00)	0.009
AS5F [15]	64.48 IQR (57.94–74.58) (12)	66.76 IQR (61.25–75.27) (2)	63.72 IQR (57.18–73.52) (10)	0.047

ESUS: Embolic Stroke of Undetermined Source; ICM: Implantable Cardiac Monitor; CPAF: Covert Paroxysmal Atrial Fibrillation; TIA: transient ischemic attack; LVEF: Left Ventricular Ejection Fraction.

**Table 2:** Comparison of ESUS patient with CPAF diagnosed on ICM and stroke patients with KIDAF. (Missing data is in brackets)

	All AF (*n* = 195)	CPAF Diagnosed on ICM (*n* = 38)	Known or Inpatient Detected AF (*n* = 157)	*p*
**Demographic data**				
Men	120/195 (61.54%)	24/38 (63.16%)	96/157 (61.15%)	0.819
Age (years)	77.00 IQR (71.00–82.00)	72.50 IQR (68.25–78.00)	79.00 IQR (72.00–84.00)	0.005
**Medical History**				
Hypertension	132/195 (67.69%)	25/38 (65.79%)	107/157 (68.15%)	0.780
Diabetes	42/195 (21.54%)	8/38 (21.05%)	34/157 (21.66%)	0.935
Dyslipidemia	83/195 (42.56%)	21/38 (55.26%)	62/157 (39.49%)	0.078
Current smoker	20/195 (10.26%)	6/38 (15.79%)	14/157 (8.92%)	0.210
Ischemic stroke/TIA	37/195 (18.97%)	11/38 (28.95%)	26/157 (16.56%)	0.081
Coronaropathy	34/195 (17.44%)	8/38 (21.05%)	26/157 (16.56%)	0.513
Peripheral arterial disease	10/195 (5.13%)	2/38 (5.26%)	8/157 (5.10%)	0.966
**Treatments on admission**				
Antiaggregant	61/195 (31.28%)	18/38 (47.37%)	43/157 (27.39%)	0.017
Anticoagulant	51/195 (26.15%)	0/38 (0.00%)	51/157 (32.48%)	<0.001
Statin	56/195 (28.72%)	15/38 (39.47%)	41/157 (26.11%)	0.102
Antihypertensive	120/195 (61.54%)	20/38 (52.63%)	100/157 (63.69%)	0.208
Beta blockers	64/195 (32.82%)	12/38 (31.58%)	52/157 (33.12%)	0.856
**Baseline stroke characteristics**				
Clinical stroke severity (NIHSS)	11.00 IQR (4.00–19.75) (5)	3.00 IQR (1.00–8.25) (2)	14.50 IQR (6.00–21.00) (3)	<0.001
Multi-territorial strokes	27/195 (13.85%)	2/38 (5.26%)	25/157 (15.92%)	0.088
Anterior circulation stroke	161/195 (82.56%)	25/38 (65.79%)	136/157 (86.62%)	0.002
Posterior circulation stroke	47/195 (24.10%)	13/38 (34.21%)	34/157 (21.66%)	0.104
Symptomatic arterial occlusion	113/188 (60.10%)	14/37 (37.84%)	99/151 (65.56%)	0.002
IV-thrombolysis (rt-PA)	60/195 (30.77%)	9/38 (23.68%)	51/157 (32.48%)	0.292
Mechanical thrombectomy	61/195 (31.28%)	5/38 (13.16%)	56/157 (35.67%)	0.007
**Biological evaluation**				
NT-proBNP (ng/L)	875.00 IQR (393.55–2 421.75) (17)	350.00 IQR (130.60–654.00) (3)	1 254.00 IQR (605.30–3 212.00) (14)	<0.001
HS Troponin T (ng/L)	21.00 IQR (12.00–34.75) (13)	17.00 IQR (10.50–26.00) (3)	23.00 IQR (13.00–36.00) (10)	0.047
TSH (mUI/L)	1.57 IQR (0.91–2.98) (27)	2.16 IQR (1.30–2.96) (3)	1.42 IQR (0.87–2.97) (24)	0.129
**ASCOD stroke subtypes**				
Atherothrombosis (grades 2–3)	126/195 (64.62%)	30/38 (78.95%)	96/157 (61.15%)	0.039
Small vessel disease (grades 2–3)	37/195 (18.97%)	7/38 (18.42%)	30/157 (19.11%)	0.923
**TTE Characteristics**				
LVEF < 60%	43/160 (26.88%)	4/38 (10.53%)	39/122 (31.97%)	0.009
Left atrial dilatation	104/151 (68.87%)	26/37 (70.27%)	78/114 (68.42%)	0.833
Left ventricular hypertrophy	61/143 (42.66%)	11/36 (30.56%)	50/107 (46.73%)	0.090
Left ventricular dilatation	10/142 (7.04%)	2/36 (5.56%)	8/106 (7.55%)	0.687
Valvulopathies	38/160 (23.75%)	9/38 (23.68%)	29/122 (23.77%)	0.991
**AF predictive scores**				
CHA_2_DS_2_-VASc [13]	5.00 IQR (4.00–5.00)	5.00 IQR (4.00–5.00)	5.00 IQR (4.00–6.00)	0.042
STAF [14]	7.00 IQR (5.00–8.00) (47)	7.00 IQR (5.00–7.00) (3)	7.00 IQR (5.00–8.00) (44)	0.655
HAVOC [16]	4.00 IQR (2.00–4.00) (35)	4.00 IQR (2.00–4.00)	4.00 IQR (2.00–4.00) (35)	0.291
AS5F [15]	75.72 IQR (69.04–82.56) (5)	66.76 IQR (61.25–75.27) (2)	76.94 IQR (70.71–83.13) (3)	< 0.001

ICM: Implantable Cardiac Monitor; CPAF: Covert Paroxysmal Atrial Fibrillation; TIA: transient ischemic attack; LVEF: Left Ventricular Ejection Fraction.

## Data Availability

Data sharing not applicable.
